# The constant threat from a non-native predator increases tail muscle and fast-start swimming performance in *Xenopus* tadpoles

**DOI:** 10.1242/bio.029926

**Published:** 2017-11-15

**Authors:** Tsukasa Mori, Yukio Yanagisawa, Yoichiro Kitani, Goshi Yamamoto, Naoko Goto-Inoue, Tadashi Kimura, Keiko Kashiwagi, Akihiko Kashiwagi

**Affiliations:** 1 The Department of Marine Biology, Nihon University College of Bioresource Sciences, Kameino 1866, Fujisawa 252-0880, Japan; 2 Cellular Glycome-targeted Technology Research Group, Biotechnology Research Institute for Drug Discovery, National Institute of Advanced Industrial Science and Technology (AIST), Ibaraki 305-8568, Japan; 3 Hiroshima University Amphibian Research Center, Higashi-Hiroshima 739-8526, Hiroshima, Japan

**Keywords:** Tadpoles, C-start, Non-native predator, Tail muscle

## Abstract

Predator-induced phenotypic plasticity is the ability of prey to adapt to their native predator. However, owing to environmental changes, encounters with unknown predators are inevitable. Therefore, study of prey and non-native predator interaction will reveal the primary stages of adaptive strategies in prey-predator interactions in the context of evolutionary processes. Here, *Xenopus* tadpoles exposed to a non-native predator, a larval salamander, showed a significant increase in body weight and tail length to body length ratio. The T_max_^2^ test indicated a significant enhancement of the tail muscle and decrease in the relative ventral fin height in tadpoles exposed to predation risk, leading to significantly higher average swimming speeds. The analysis of muscle-related metabolites revealed that sarcosine increased significantly in tadpoles exposed to non-native predators. Multiple linear regression analysis of the fast-start swimming pattern showed that the fast-start swimming speed was determined by the time required for a tadpole to bend its body away from the threat (C-start) and the angle at which it was bent. In conclusion, morphological changes in tadpoles were functionally adaptive and induced by survival behaviors of *Xenopus* tadpoles against non-native predators.

## Abstract (precis)

**Summary:** *Xenopus* tadpoles exposed to a non-native predator increase their tail length, muscle tissue and swimming speed, all of which are functionally adaptive and induced by tadpole survival behavior.

## INTRODUCTION

Phenotypic plasticity is the ability to produce different phenotypes in response to changes in environmental conditions, thus facilitating adaptation to different environmental conditions (Schoeppner and Relyea, 2005; Spitze, 1992; Trussell, 1996). An inducible defense stimulated by a predation threat is a type of phenotypic plasticity (Bronmark and Miner, 1992; Gilbert, 2009; Jarrett, 2009; Kishida and Nishimura, 2004; Tollrian, 1995; Tollrian and Harvell, 1999; Weisser et al., 1999). Anuran tadpoles are suitable models of predator-induced phenotypic plasticity, many of which display a heightened tail depth phenotype against various types of predators, including dragonfly larvae (Kishida et al., 2010; McCollum and Leimberger, 1997; Van Buskirk et al., 1997). For example, a bulgy morph formation was observed in *Rana pirica* tadpoles after their exposure to their main predator, the larval salamander *Hynobius retardatus*, which swallows tadpoles live (Kishida and Nishimura, 2004). These predator-induced morphological changes in tadpoles affect their performance; for example, they may increase their swimming speed to evade predators (Johnson et al., 2015). Therefore, tadpoles displaying phenotypic plasticity show higher survival rates when placed with native predators (Kishida and Nishimura, 2005; Van Buskirk and Relyea, 1998). These adaptive strategies involving phenotypic plasticity are found in tadpoles as a defense mechanism against specific predators to enhance their ability to escape predators and thus increase their chance of survival in prey-predator interactions occurring in nature.

Exposure to new and previously unknown predators may equally threaten the survival of tadpoles, even if they have not co-inhabited and co-adapted in a prey-predator relationship. Encountering an unknown predator resulting from environmental changes might initiate an evolutionary adaptation in prey against the new predators. This might be an early stage of adaptation in the prey-predator interaction and part of the evolutionary process that has been poorly investigated. Predation risk engendered by a threat from a non-native predator may serve as a driving force for phenotypic adaptations to the new predator. Therefore, the present experiment brought together two species that have not previously co-existed in nature.

In this experiment, we focused on the prey-non-native predator interaction between tadpoles of *Xenopus laevis*, a species that occurs naturally in southern Africa and has been used widely as a model organism in biological studies (Tinsley and Kobel, 1996), and the larval salamander, *H. lichenatus*, which is endemic to the Tohoku area of Japan. We examined whether *X. laevis* tadpoles use general adaptive strategies observed in *R. pirica* and other tadpoles when encountering the unknown predator.

Our primary aim was to examine whether the non-native predator *H. lichenatus* elicits a fear response in *X. laevis* tadpoles and whether tadpoles respond to this fear with morphological changes, such as increased tail length and height. Furthermore, the effectiveness of the changes was examined by measuring muscle-related metabolites to predict muscle volume, which is directly associated with escape behavior. In fact, various physical systems must change to increase muscle volume for increased swimming speed in tadpoles. Angiogenesis is an important factor involving various physical phenomena, including muscle development, cancer cell growth and wound healing. Resent genome research revealed that the *Xenopus* genome encodes more than 20,000 protein-coding genes, including orthologs of at least 1700 human disease genes, accounting for 79% of human diseases (Hellsten et al., 2010). Therefore, *Xenopus* may become a useful human disease model. In this report, we discussed a physiological similarity observed in *Xenopus* tadpoles caused by predation stress to that of human beings using metabolic analysis.

Various phenotypic changes have been documented in tadpoles exposed to native predators. First, when the predator was a piscivorous fish, *R. dalmatina* and *Lithobates pipiens* tadpoles showed reduced body weight, enlarged tails and increased swimming speeds (El Balaa and Blouin-Demers, 2013; Teplitsky et al., 2005). Second, when the predator was a dragonfly larva, *Pseudacris triseriata*, *Hyla versicolor* and *R. dalmatina* tadpoles showed increased body weight and enlarged tails (Van Buskirk et al., 1997), but their swimming speed did not increase (Teplitsky et al., 2005; Van Buskirk and McCollum, 2000). Third, when the predator was a larval salamander, the body of *R. pirica* tadpoles became bulgy (Kishida and Nishimura, 2004) and their tail enlarged, which could result in reduced swimming speed.

To categorize the behavior of *Xenopus* tadpoles against non-native predators, we investigated whether the swimming speed of tadpoles is influenced by morphological changes in the tadpoles. We filmed the swimming behavior of *Xenopus* tadpoles as they waved their tails after an electric shock and measured the swimming speed of the tadpoles exposed to predation and the control group.

We also analyzed the C-start response in relation to their swimming speed. The C-start is an initial fast arching of the body triggered by sudden visual or mechanosensory stimuli in fishes (Eaton et al., 1977; Preuss and Faber, 2003) and tadpoles (Sillar and Robertson, 2009). The subsequent fast-start swimming performance is considered important for successful predator avoidance (Walker et al., 2005). However, the relationship between the C-start and subsequent fast-start swimming has yet to be elucidated in tadpoles. In the present experiment, we analyzed possible explanatory variables of the C-start, i.e. the time required for a tadpole to bend its body and the angle at which it bends during the C-start, and the fast-start swimming speed to investigate the significance of the increased body weight of *Xenopus* tadpoles exposed to non-native predation threats.

## RESULTS

**Fig. 1.**
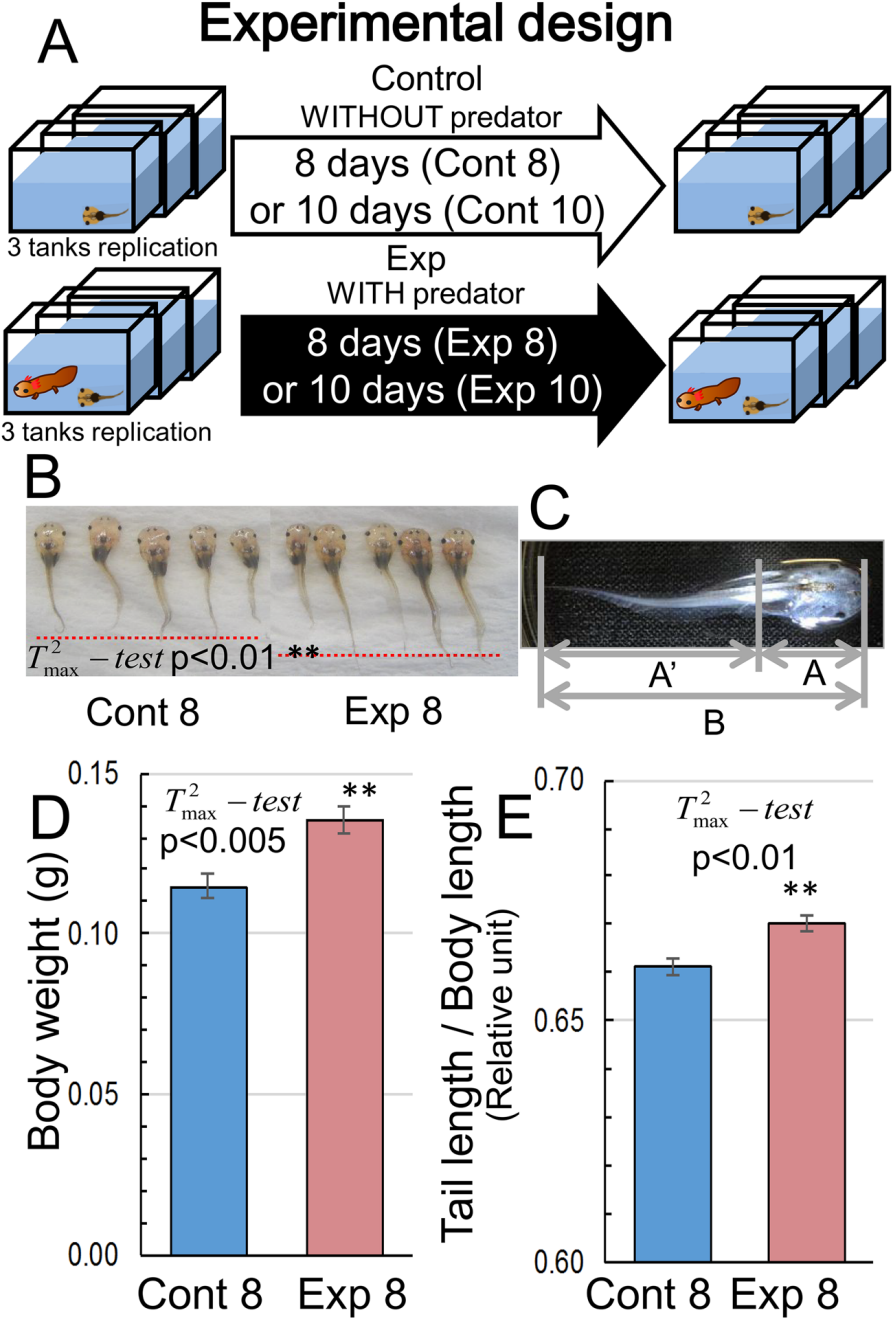
**Experimental design and changes in body length and weight in tadpoles exposed to predation.** (A) Experimental design of the induced predation in *Xenopus* tadpoles. *Xenopus* tadpoles (50 tadpoles per aquarium) were assigned to five different treatment groups. The control group (Cont) was not exposed to the predator for 8 or 10 days of the experiment. The Exp 8 and Exp 10 groups were exposed to the predator for the full 8 and 10 days, respectively. (B) Body length in *Xenopus* tadpoles exposed to predation. (C) Morphology of *Xenopus* tadpoles. Tail length was determined as the distance from the anus to the tail top. (D,E) Body weight (D) and the ratio of tail length to body length (E) in tadpoles exposed to predation for 8 days. The number of tadpoles in Cont 8 and Exp 8 was 148 and 97, respectively. Statistical analysis was performed using Krishnaiah’s 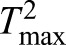-test.

In *Xenopus* tadpoles exposed to non-native predators, the mean vectors of the body weight, body length, and ratio of tail length to body length were significantly changed (approximate 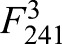
=8.015 from Pillai's statistic, *P*=0.00004). The mean body length and the mean body weight increased significantly (*P*<0.01 and *P*<0.005, respectively, according to Krishnaiah's 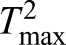) in the presence of the non-native predator (Fig. 1B,D). The increase in body length was the result of a significant increase (*P*<0.01 according to 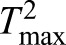) in the ratio of tail length to body length (Fig. 1C,E).

**Fig. 2.**
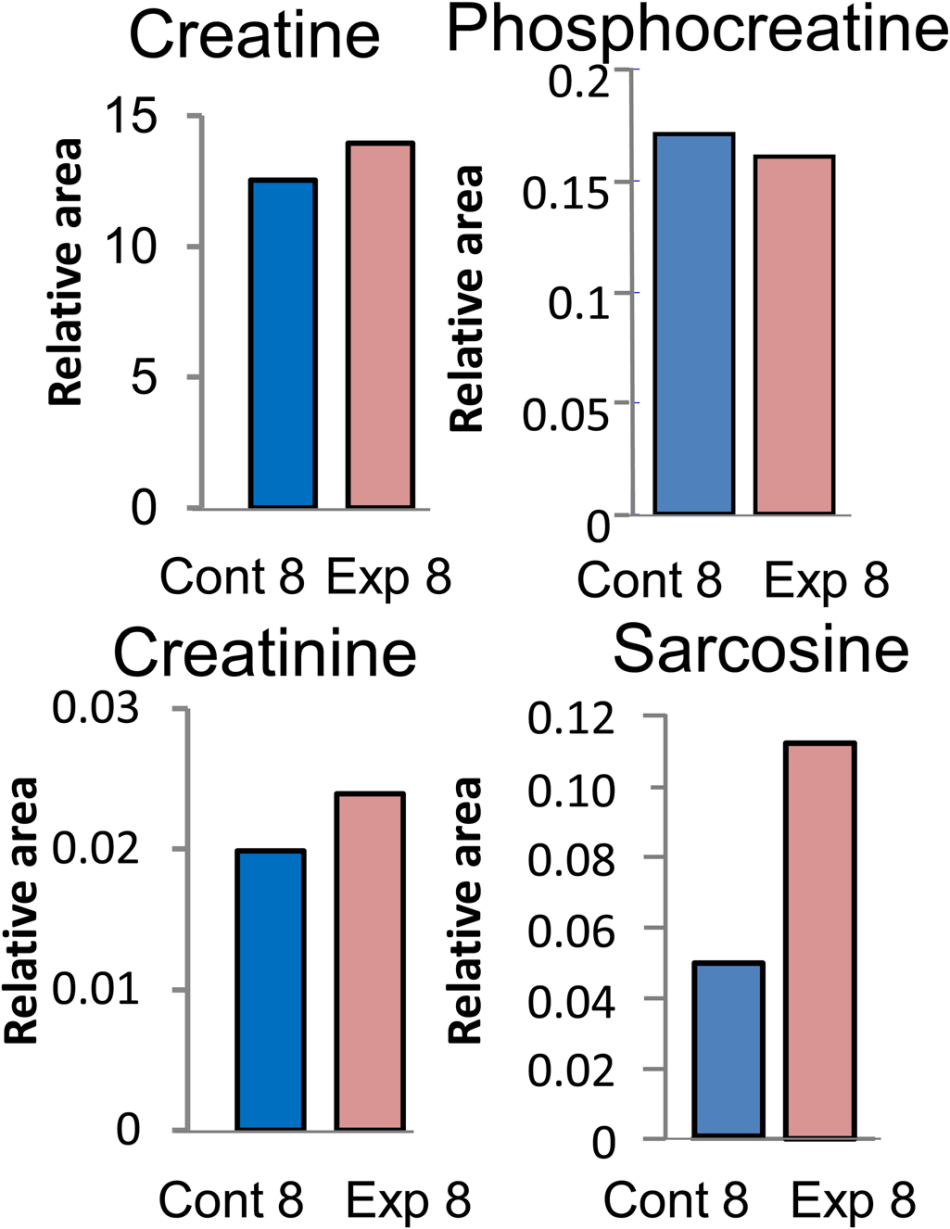
**Analysis of muscle-related metabolites using CE-TOFMS.** Ten different *Xenopus* tadpoles were randomly selected from the group of tadpoles exposed to predation for 8 days (Exp 8) and the control group (Cont 8). Then, the tadpoles were frozen in liquid nitrogen and metabolites extracted with methanol. A pool of methanol extracts from 10 tadpoles was treated as one sample and analyzed using CE-TOFMS.

**Fig. 3.**
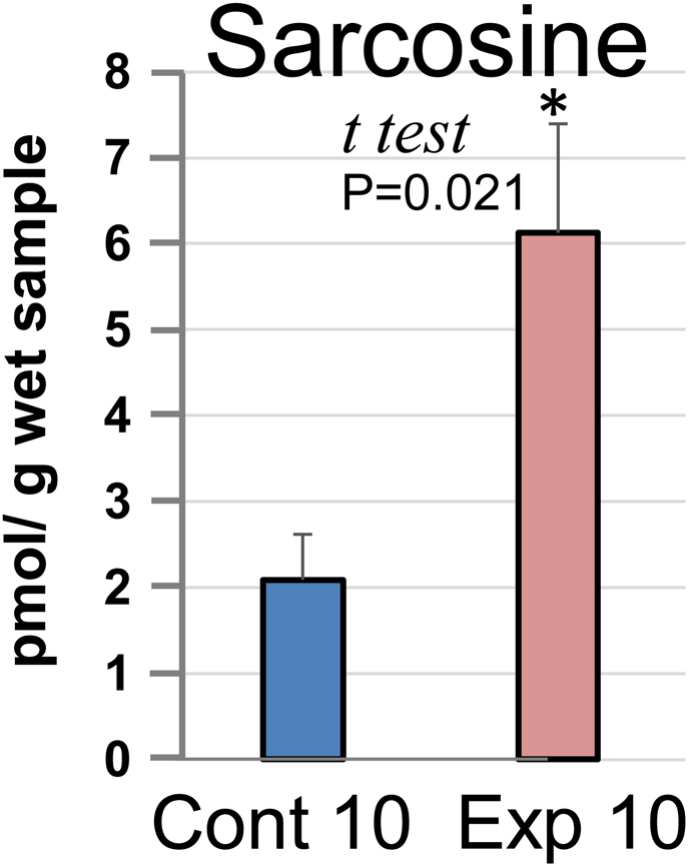
**Analysis of sarcosine using GC-MS.** Tails from nine different *Xenopus* tadpoles were randomly selected from the group of tadpoles exposed to predation for 10 days (Exp 10) and the control group (Cont 10), and three tails were combined to prepare three samples for each experiment. Sarcosine was extracted and the content was expressed as picomol of sarcosine per gram of wet sample. Statistical analysis was performed using a two-sample *t-*test.

Creatine and creatinine, one of muscle-related metabolites, increase with increasing muscle volume in mammals (Patel et al., 2013; Wyss and Kaddurah-Daouk, 2000) and frogs (Plaghki and Marechal, 1976). The analysis of CE-MS revealed that the exposure of tadpoles in Exp 8 to predation increased the creatine, creatinine and sarcosine contents by 1.1-, 1.2- and 2.2-fold, respectively, compared with that in the control (Fig. 2). In contrast, the phosphocreatine level was 0.9-fold lower after exposure to predation than that in the control. For sarcosine content, neither the Kolmogorov–Smirnov nor *F*-test was significant at the 5% level; therefore, we performed a one-tailed *t*-test under the assumption of homogeneity of variance. We chose the one-tailed test because we suspected an increase in sarcosine levels from Cont 10 to Exp 10. The *P*-value for the *t*-test was 0.021, and sarcosine content was increased significantly (*P*<0.05) in tadpole tails from Exp 10 compared to that in the control (Fig. 3).

**Fig. 4.**
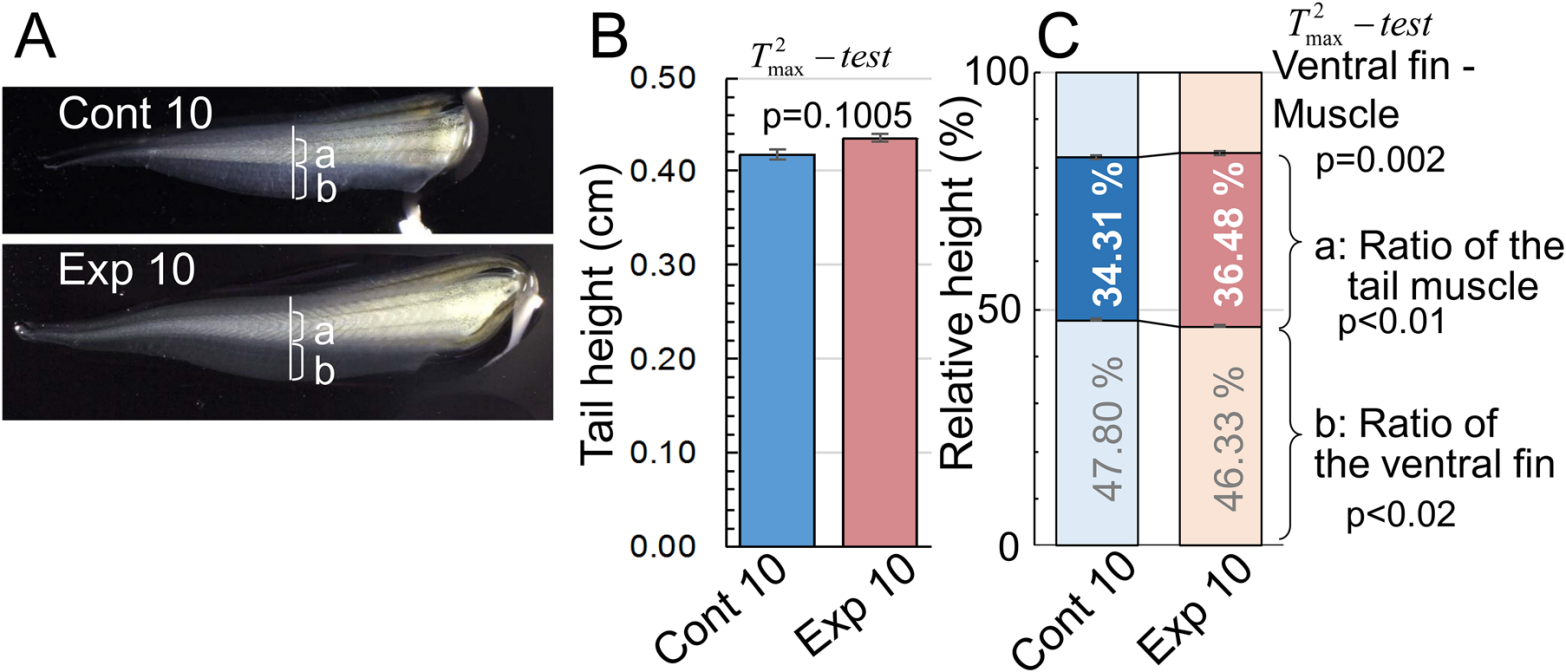
**Measurements of tail height, tail muscle and height of the ventral fin.** (A) Photographs of tadpoles in the control (Cont 10) and experimental group (Exp 10). Lines indicate tail height, and the lowercase letters a and b indicate height of the tail muscle and height of the ventral fin, respectively. Tail height (B), height of the tail muscle, and height of the ventral fin (C) were measured in 50 tadpoles from each group of tadpoles exposed to predation for 10 days (Exp 10) and the control (Cont 10). These values were obtained at the points of the greatest height of tadpole's tail from images of tadpoles using ImageJ software.

To validate and clarify the induced tadpole phenotype, i.e. muscle tissue enhancement from predation exposure, we extended the predation exposure to 10 days (Fig. 1A). The extended exposure produced similar results to those obtained for the Exp 8 and Cont 8 groups: the mean vectors of the relative tail muscle, relative ventral fin height, and tail height between Cont 10 and Exp 10 were significantly different (approximate 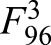
=8.547 from Pillai's statistic, *P*=0.00004). There was no significant difference between Cont 10 and Exp 10 in tail height (*P*=0.1005 according to 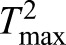) (Fig. 4A), whereas the tail muscle height was increased significantly (*P*<0.01 according to 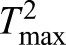) in Exp 10 (Fig. 4B,C). The ventral fin height was decreased significantly (*P*<0.02 according to 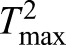), although the fin was expected to enhance the swimming ability. We noted that, according to Krishnaiah's 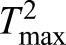 statistic, a 99.8% confidence interval for {*μ*(*ventral*, *Cont*10)−*μ*(*muscle*, *Cont*10)}−{*μ*(*ventral*, *Exp*10)−*μ*(*muscle*, *Exp*10)} was (0.0000, 0.0729), where *μ*(*ventral*, *Cont*10), *μ*(*muscle*, *Cont*10), *μ*(*ventral*, *Exp*10), and *μ*(*muscle*, *Exp*10) were the mean relative ventral fin height for Cont 10, the mean relative muscle height for Cont 10, the mean relative ventral fin height for Exp 10, and the mean relative muscle height for Exp 10, respectively.

**Fig. 5.**
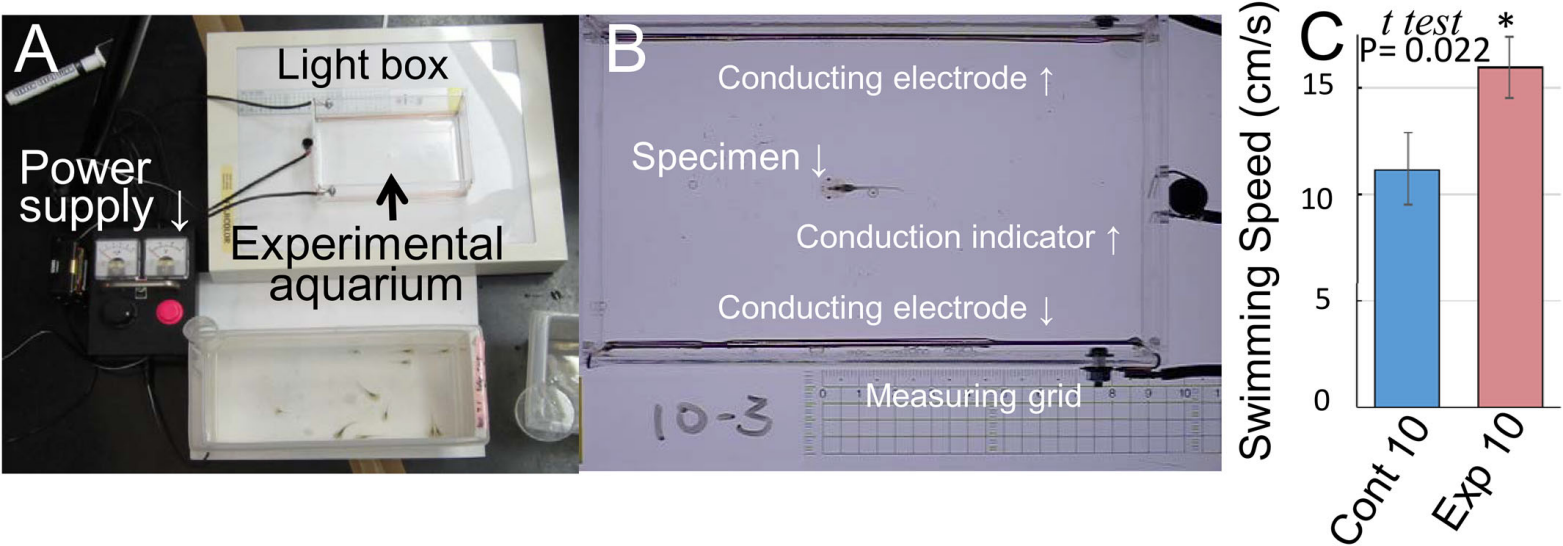
**Swimming performance test.** Ten tadpoles were randomly chosen from each experimental aquarium, and each tadpole was placed into an experimental electric chamber (A,B). After stabilizing a tadpole's behavior, an electric shock (12 V, 1.5 mA) was delivered to the tadpole for ∼300 ms (B). Swimming behavior of the tadpole was filmed with a high-speed camera at 240 frames/s (Movie 1), and the fast-start swimming speed was analyzed using tadpoles from the control (Cont 10) and the group of tadpoles exposed to predation for 10 days (Exp 10) (C). Statistical analysis was performed using a two-sample *t-*test after the Kolmogorov–Smirnov test.

For precise measurements of the swimming speed (at the millisecond level), we prepared an experimental electric chamber (Fig. 5A) and ensured that a tadpole inside the chamber did not start swimming before the electric shock, as shown in Fig. 5B. Since neither the Kolmogorov–Smirnov nor the *F* test was significant at the 5% level, we performed the one-tailed *t*-test assuming homogeneity of variances. We found that the swimming speed increased when tadpoles were exposed to a constant predation threat (*P*=0.022 for one-tailed *t*-test) (Fig. 5C), i.e. continuous stress increased the swimming speed of tadpoles, which is what we suspected and why a one-tailed *t*-test was performed.

**Fig. 6.**
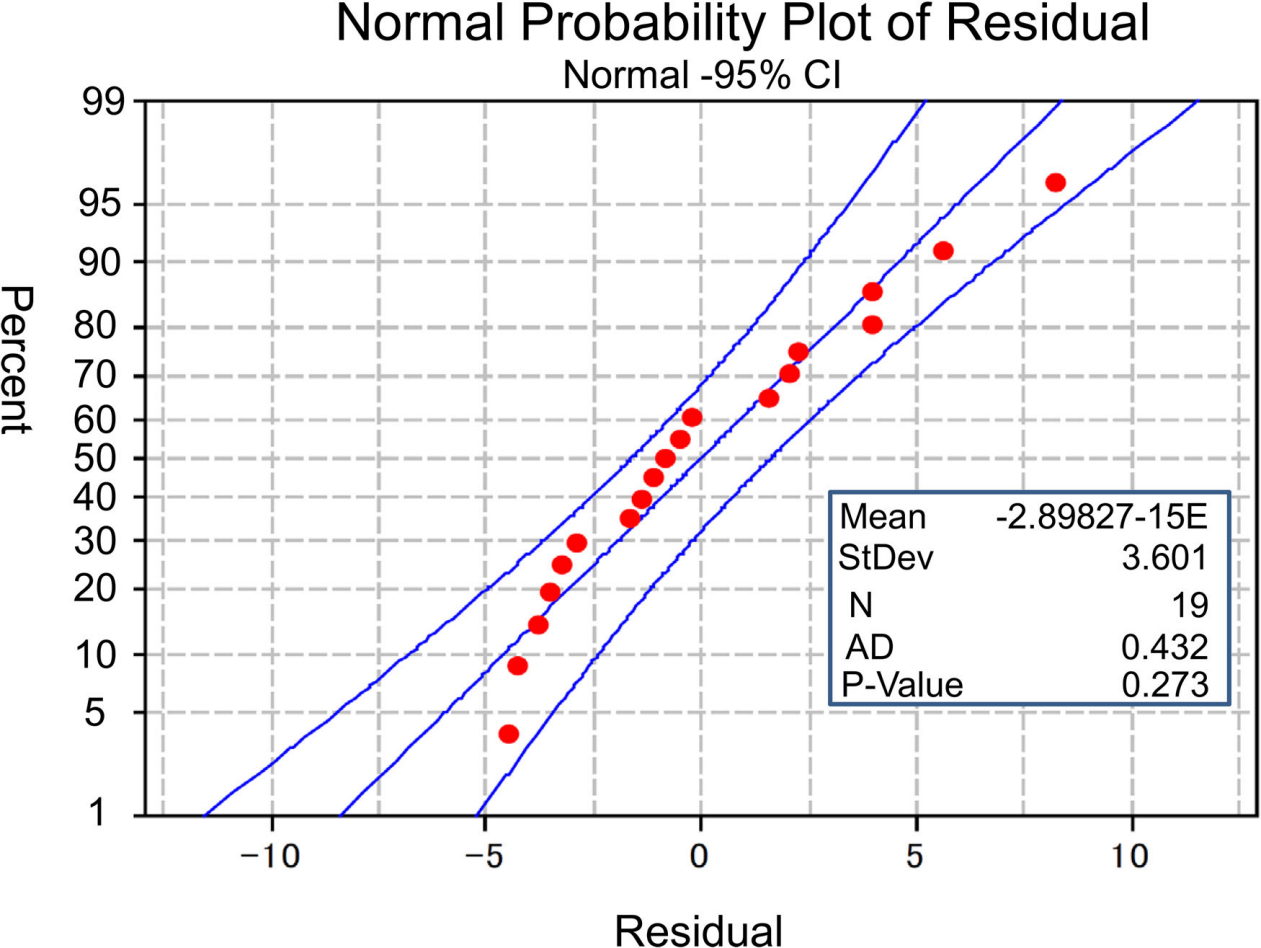
**Normal probability plot for the residual of the regression analysis of the fast-start swimming speed.** All points of the normal plot for the residual were within a 95% confidence interval (CI, confidence interval; N, the number of residuals; AD, Anderson-Darling statistic; St Dev, standard deviation of residuals).

The best model for the multiple linear regression analysis of the fast-start swimming speed observed just after delivering the electric shock was tested by three different methods, i.e. the stepwise model selection method, 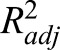 and Akaike information criterion; all three methods selected the same model. Careful examination of the residual plots revealed no further explanatory variables, and none of the assumptions of the regression analysis were violated at the 5% significance level (see Fig. 6), indicating that the model selected was reasonable. The selected model was *y*=10.909−0.09855*x*_1_+0.06922*x*_2_ , where *y*, *x*_1_ and *x*_2_ are the fast-start swimming speed, the time required for a tadpole to bend its body to swim away (C-start), and the angle in degrees at which a tadpole bends its body when assuming the position for C-start, respectively (Table 1). The ANOVA results are shown in Table 2, and the regression coefficients and their *P*-values are shown in Table 1. The adjusted coefficient of determination was 
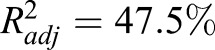
, which showed that the model explained almost 50% of the variation in the observed fast-start swimming speed (Table 2).

**Table 1.**
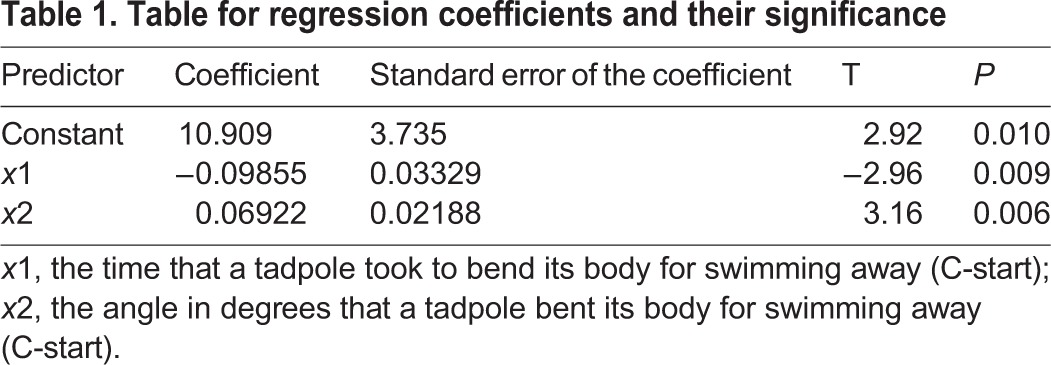
**Table for regression coefficients and their significance**

**Table 2.**
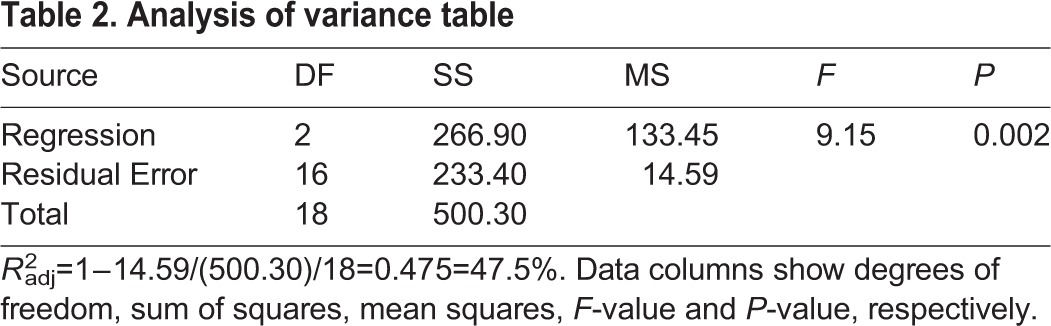
**Analysis of variance table**

## DISCUSSION

It is well known that predator-induced phenotypic plasticity is the result of adaptation to predators, and many reports have shown that variations in morphological features in relation to various predators are the product of adaptive evolution during the prey–predator interaction as described in the Introduction. *Xenopus* tadpoles exposed to the non-native predator larval salamander exhibited several morphological changes, including tail elongation and increased body weight (Fig. 1D,E). Moreover, the levels of creatine, creatinine, and sarcosine increased in *Xenopus* tadpoles (Fig. 2), suggesting that the muscle mass of tadpoles experiencing predation fear was greater than that of the control. Creatine is a basic energy reserve in the fast-twitch muscle fiber, where it is converted in the presence of creatine kinase into phosphocreatine, an immediate source of energy used to replenish ATP (Wyss and Kaddurah-Daouk, 2000). Creatine kinase was detected previously in *Xenopus* tadpoles (Gillardin et al., 2009) and, therefore, the presence of phosphocreatine was expected, as shown in Fig. 2. Large pools of phosphocreatine are present in fast-twitch skeletal muscles for immediate ATP regeneration during short periods of intense muscle contraction (Wyss and Kaddurah-Daouk, 2000). As shown in Fig. 2, tadpoles exposed to predation showed a lower tendency for phosphocreatine accumulation, whereas the creatine level in Exp 8 was greater than that in Cont 8. This implies that the consumption of phosphocreatine during muscle contraction was greater in tadpoles subjected to predation than in the control. Furthermore, muscular creatine and phosphocreatine are converted to creatinine (Wyss and Kaddurah-Daouk, 2000), the serum levels of which have been used as a marker of muscle mass in various studies (Patel et al., 2013). Therefore, increased levels of creatinine and creatine suggested that the muscle mass of tadpoles experiencing predation fear was greater than that of the control.

The relative content of sarcosine was 2.2-fold higher in the group subjected to predation than in the control. Sarcosine is produced by different pathways: from creatine in the gut (Roberts and Walker, 1985) or from dimethylglycine in mitochondria (Alsayed et al., 2013). Produced sarcosine is then degraded to glycine or used for creatine synthesis (Wyss and Kaddurah-Daouk, 2000). However, recent reports identified sarcosine as a marker of prostate cancer, since the knockdown of the enzyme that generates sarcosine from glycine attenuated prostate cancer cells (Sreekumar et al., 2009).

Furthermore, sarcosine influences endothelial cell function in angiogenesis through the modulation of the PI3K/Akt/mTOR pathway (Sudhakaran et al., 2014). Additionally, elevated levels of dimethylglycine are involved in congenital heart defects, although the mechanisms remain unknown (Alsayed et al., 2013). In the present study, the amount of *N,N*-dimethylglycine was 1.4-fold greater in tadpoles exposed to predation than in the control (data not shown). Therefore, sarcosine production from dimethylglycine might be correlated with predation fear. Since the role of sarcosine is somewhat different from the role of other muscle-related metabolites, we re-analyzed the content of sarcosine in tadpole tails from Cont 10 and Exp 10. Sarcosine levels in tadpole tails of Exp 10, a group that showed increased tail muscle, were about threefold greater than those of the control (Fig. 3). Enhancement of skeletal muscle through PGC-1α is correlated with angiogenesis in mammals (Tadaishi et al., 2011). Therefore, the enhanced tail muscle height may be owing to increased angiogenesis rates (Fig. 4). In future research, it is necessary to clarify the relationship between fear stress, muscle development and angiogenesis, e.g. whether the high sarcosine levels during fear stress are responsible for muscle development and angiogenesis, which could help to develop treatments for human diseases such as sarcopenia.Furthermore, in this experiment, *Xenopus* tadpoles exposed to predation fear increased their swimming speed (Fig. 5). There are many reports concerning the adaptive strategy and its effectiveness against predators in relation to the prey-predator interaction in nature. Our results indicate that fear, excluding ecological background, stimulates the survival instinct and enhances the fin muscle, resulting in increased swimming speed.

The effectiveness of fast-start swimming as a means of evading predators has been studied extensively, using fish species as predators. Thus, anuran tadpoles in the presence of fish predators develop small bodies, long tails and large tail muscles to increase fast-start swimming (Johnson et al., 2015; Teplitsky et al., 2005). In contrast, high tails in tadpoles, such as in *R. dalmatina* and *H. versicolor*, induced by dragonflies do not improve fast-start swimming performance (Teplitsky et al., 2005; Van Buskirk and McCollum, 2000). However, tadpoles such as those of *R. pirica* are less vulnerable to predation by larval salamanders (*H. retardatus*) than non-induced tadpoles (Kishida and Nishimura, 2005).

*Xenopus* tadpoles increase body weight but do not show the bulgy phenotype observed in *R. pirica* tadpoles in response to their main predator, larval salamander *H. retardatus* (Kishida and Nishimura, 2004). Large tadpole size is also an effective tactic against smaller invertebrate predators (Cronin and Travis, 1986). These data indicated that the increase in body weight, tail length and tail height, as a strategy of *Xenopus* tadpoles against the non-native predator *H. lichenatus*, is neither that towards piscivorous fish nor invertebrate predators.

Some reports indicate that fast-start swimming, which consists of the C-start (fast body bending) followed by fast beating of the tail, is highly related to survival probability in fishes. However, there are no reports on the relationship between the swimming speed and C-start. Anuran tadpoles also increase their probability for survival by fast-start swimming in the presence of fish predators (Johnson et al., 2015; Teplitsky et al., 2005); similar escape mechanisms by swimming were observed in *X. laevis* tadpoles (Sillar and Robertson, 2009). Although studies on the fast-start swimming speed in tadpoles have been conducted, the relationship between the C-start and subsequent fast-start swimming has yet to be elucidated. The regression analysis showed that the swimming speed of tadpoles was determined by the time it takes a tadpole to bend its body and the angle at which the body is bent (Table 1). The model explained almost 50% of the variation (the bending speed and angles of tadpoles) in the observed fast-start swimming speed, indicating a possible strategy of *Xenopus* tadpoles against non-native predators. As described above, increase in body weight and body size might be ineffective tactics for escaping piscivorous fish, since these changes do not increase the swimming speed of tadpoles. However, this increase in body weight and size might heighten the centrifugal force of the C-start, and thus increase the chances for escaping from salamander or invertebrate predators (Cronin and Travis, 1986). These results indicate that *Xenopus* tadpoles maintain the C-start without decreasing swimming speed.

Our study shows that when salamander is a non-native predator, *X. laevis* tadpole body weight increases slightly, the tail becomes larger, and its swimming speed increases. This indicates that *Xenopus* tadpoles assume a flexible strategy against the unknown predator, which is different from that against piscivorous fish, invertebrate predators and salamanders. *Xenopus* tadpoles employ different strategies to survive in the presence of a new predator, whereby they develop a new strategy, which explains why the regression model explains only 50% (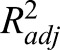) of the variation in swimming speed. In contrast, if a tadpole faces a native predator, it adopts a suitable strategy, which should explain more than 50% of the variation in the strategy by adequate explanatory variables.

## MATERIALS AND METHODS

### Ethics statement

All animal experiments were conducted by trained personnel in accordance with the guidelines of the Animal Care Committee, Nihon University.

### Experimental animals and setup

*Xenopus laevis* tadpoles of the J strain at stage 51 were obtained from a commercial dealer at Watanabe Zoushyoku (Hyogo Prefecture, Japan). The tadpoles were fed boiled and pureed green peas (2 g) every 2 days during the experimental period. Larval salamanders (*H. lichenatus*) were purchased from a commercial dealer (Toshin Co., Ltd., Ogaki, Japan). They were kept in 2-L aquaria and fed *Tubifex ad libitum* until their bodies reached a length of ∼4 cm. Tap water treated with activated charcoal (Tsurumicoal) for 24 h was used in this experiment. The experiment was conducted in a laboratory under a natural day/night regime (∼14/10 h) at 20°C. Groups of 50 similarly sized tadpoles were randomly collected from a 20-L holding tank and placed in 2.5-L aquaria (25×10 cm, height 10 cm) containing 2 L of treated tap water. To collect the tadpoles, we generated two sets of uniform pseudo-random numbers, i.e. the first set included numbers between 1 and 6 and the second set included numbers between 1 and 50. At the top of each tank, the surface area was roughly divided into six parts labeled 1 to 6. The number of *Xenopus* tadpoles in each area varied at any time of the experiment. The uniform pseudo-random number generated in the first set determined the number of the sampling area. The uniform random number generated in the second set identified the *Xenopus* tadpole counted from left to right. The aquaria were then divided into control (Cont) and treatment (Exp) groups. We prepared two sets of experiments: tadpoles were exposed to the predator for 8 days (Cont 8 and Exp 8) or 10 days (Cont 10 and Exp 10) (Fig. 1A). In the 8-days experiment, Cont 8 and Exp 8 tadpoles were examined and compared to determine any morphological changes that might have occurred under the non-native predation threat, including changes in body weight, tail length, and metabolite levels. In the 10-days experiment, sarcosine content in the tail tissue was measured using gas chromatography–mass spectrometry (GC-MS), and the swimming performance of the Cont and Exp tadpoles was examined to determine whether any such phenotypic changes enhanced the escape mechanism of tadpoles exposed to predation. For all the experiments, we prepared two sets of aquaria, each consisting of three main and three backup aquaria; the experimental conditions (exposure to predation, temperature, aeration, etc.) in the backup aquaria were the same as those in the main aquaria. The experiments were initiated at the same time and the number of surviving tadpoles was counted in each aquarium daily. In the Exp 8 and Exp 10 groups, any tadpoles lost to predation by the salamander or moribund tadpoles were replaced by randomly selected tadpoles from the backup aquarium (also reared in the presence of the salamander) to maintain a minimum of 50 tadpoles per aquarium in each treatment and thus eliminate any bias in experimental results owing to density effects.

### Conceptual experimental design of predator-induced fear stress

If we distinguish between the causes of fear, e.g. odor, sight, hearing, physical contact, etc., we may be able to detect an effect if we combine several causes, but if the causes are examined individually, we may fail to identify the same effect. Since there are several causes of fear, this protocol is equivalent to the process of identifying the treatment effect using MANOVA. We provided a hypothetical example where we could identify the treatment effect using bivariate data, but were not able to find the treatment effect if we used a single variate under a bivariate scenario. We assumed the following scenario:(*x*, *y*) are (1, 3), (2, 2) and (3, 1) for treatment level 1, and (3, 5), (4.1, 4) and (5, 3) for treatment level 2; *μ*_*x*,1_, *μ*_*x*,2_, *μ*_*y*,1_ and *μ*_*y*,2_ are the mean of *x* for treatment level 1, the mean of *x* for treatment level 2, the mean of *y* for treatment level 1, and the mean of *y* for treatment level 2, respectively. The variables *x* and *y* represent the effect of the vision and the effect of the odor, respectively, assuming that we can measure these effects. Pillai's trace is 0.99973 and the *P*-value based on Pillai's trace is 0.0000, i.e. it is highly significant. The 95% confidence intervals are − 6.16<*μ*_*x*,1_−*μ*_*x*,2_<2.09, − 6.12<*μ*_*y*,1_−*μ*_*y*,2_<2.12 and − 4.20<(*μ*_*x*,1_−*μ*_*x*,2_)+(*μ*_*y*,1_−*μ*_*y*,2_)<− 3.87. If a confidence interval for the difference between two means includes 0, then they are not significantly different; otherwise their difference is significant. Thus, the treatment effect is not significant at 5% level if we consider *x* or *y* individually, but it becomes significant at 5% if both *x* and *y* are considered together. With the extension from bivariate to multivariate, i.e., from two to several causes, the confidence interval could be the mean of the linear combination of all the causes (e.g. the summation of the means of all the causes). Therefore, we combined the causes of fear in our experimental design.

### Measurements of body weight, body length and tail height

At the end of the experiments, the tadpoles were transferred individually to a small glass case containing rearing water and photographed with a camera to measure the body parts. The length of each body part was analyzed using ImageJ software (https://imagej.nih.gov/ij/). The measured tadpoles were then placed on Kimwipes to eliminate surface water and their body weight was measured using an electronic scale. The heights of the tail, tail muscle and ventral fin were measured on 50 tadpoles from each Exp 10 and Cont 10. These values were obtained at the points of greatest tail height using Image J software. Data on tail and muscle height are available in Table S1.

### Capillary electrophoresis time-of-flight mass spectrometry (CE-TOFMS) analysis

Ten different tadpoles of similar size (130–140 mg in body weight) were obtained from each Exp 8 and Cont 8. Each tadpole was placed on a Kimwipe to remove the surface water, and its body weight was measured. The tadpoles were anesthetized in ice water, then frozen in liquid nitrogen, and immediately placed in methanol (500 μl) containing internal standards (50 μM internal standard H3304-1002; Human Metabolome Technologies, Tsuruoka, Japan). After freezing in liquid nitrogen to inactivate the enzymes, each tadpole was homogenized five times at 4000 rpm for 60 s using a bead cell disrupter (MS-100R; TOMY Digital Biology, Tokyo, Japan). The samples were then centrifuged at 2300×***g*** for 5 min at 4°C, and 50 μl of each supernatant from 10 tadpoles were pooled separately for Exp 8 and Cont 8 and transferred to a new tube. Each pooled sample (500 µl) was diluted with 200 μl Milli-Q water and 500 μl chloroform, thoroughly mixed, and centrifuged at 2300×***g*** for 5 min at 4°C. The upper aqueous layer (400 μl) was centrifugally filtered through a Millipore 5 kDa cut-off filter to remove proteins. The filtrate was lyophilized and suspended in 50 μl Milli-Q water and analyzed by CE-TOFMS (Soga et al., 2003). The CE-TOFMS was carried out in a CE Capillary Electrophoresis System equipped with a 6210 Time of Flight mass spectrometer, 1100 isocratic HPLC pump, G1603A CE-MS adapter kit and G1607A CE-ESI-MS sprayer kit (Agilent Technologies, Waldbronn, Germany). The system was controlled by G2201AA ChemStation software version B.03.01 for CE (Agilent Technologies). Data (nmol g^−1^ sample) were expressed as values of the relative area analyzed by the peak of the CE-TOFMS using the automatic integration software MasterHands ver.1.0.6.12 (Sugimoto et al., 2010).

### Sarcosine analysis using GC-MS

The collection of the samples, including the extraction and derivatization of low-molecular-weight metabolites, was performed as follows. Briefly, three tadpole tails were collected from Exp 10 and Cont 10 and homogenized in 625 μl extraction solution (MeOH:H_2_O:CHCl_3_, 1:1:0.5) to extract metabolites. After centrifugation at 16,000×***g*** for 5 min at 4°C, the supernatant was collected and diluted with 200 μl H_2_O. The solution was mixed and centrifuged at 16,000×***g*** for 5 min at 4°C, and 250 μl of the resultant supernatant was transferred to a clean tube and dried. For derivatization of free amino acids, we followed the manufacturer's protocol for EZ:faast free amino acid analysis by GC-MS (Phenomenex, Torrance, CA, USA) and performed the GC-MS analyses in a 5977B GC/MSD system (Agilent Technologies). In this system, sarcosine and an internal standard (norvaline) that was added to the sample were separated using GC-MS, and the sarcosine content was determined by comparing the peak area against the internal standard and expressed as picomol per gram of wet sample (Fig. 3).

### Swimming performance test

Ten tadpoles were randomly chosen following the protocol described above from each experimental aquarium, and each tadpole was placed into an experimental electric chamber (length: 15 cm, width: 10 cm, height: 5 cm), which contained 150 ml water. After stabilizing the tadpole's behavior, it was given an electric shock (12 V, 1.5 mA) for ∼300 ms. Appropriate voltage and current to stimulate tadpoles were determined by experimenting on tadpoles from the backup aquariums.

The swimming behavior of the tadpole was filmed with a high-speed camera (CASIO EX-FC150, Tokyo, Japan) at 240 frames s^−1^ (Movie 1). The film was analyzed using ImageJ software to determine the correlation between predation and the following data: body length, tail length, body width, tail width, rotation time, rotation angle, time required for three tail beatings, distance a tadpole covers during three tail beatings, swimming speed during three tail beatings, time required until tadpole stops moving, and distance a tadpole passes during that time. Data on swimming speed are available in Table S2.

### Statistical methods

Statistical differences in body weight, body length, and the ratio of tail length to body length in tadpoles from the Exp 8 and Cont 8 groups were analyzed using Pillai's test of one-way multivariate analysis of variance (MANOVA) with two levels. Since the repetition of single variate tests for multivariate data will increase the level of significance, it is therefore inappropriate to repeat single variate tests. The same test was also used to compare the relative ventral fin height and muscle height between tadpoles from the Exp 10 and Cont 10 groups. Pillai's test is more robust than other tests, including Wilk's lambda, when the violation of normality and/or homogeneity of the variance-covariance matrix is met (Olson, 1974). Since the application of the multiple comparison to multivariate data allows the pairwise significance difference among the mean vectors to be identified, the two sets of data were further analyzed by Krishnaiah's 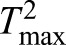 statistic for estimating the simultaneous confidence interval for multiple comparisons of the difference between two mean vectors (Subbaiah and Mudholkar, 1981). We investigated whether the fast-start swimming speed of Cont 10 and that of Exp 10 followed normal distribution using the Kolmogorov–Smirnov test. If the assumption of normality was not violated, we performed the homogeneity of variances test based on the *F* distribution. Depending on the results of the homogeneity of variances test, we applied either Welch's *t*-test, when heterogeneity of variances was met, or the two-sample *t*-test, if the equivalence of means under homogeneity of variances was assumed, which is uniformly the most powerful test. We noted that *t*-tests are robust for the departure from the assumption of normality as far as the normality assumption is not significantly violated at a given significance level of the test (see Rao, 1973 and Hampel, 2000). If the assumption of normality was violated, the Mann–Whitney nonparametric test was performed. Moreover, we applied the multiple linear regression analysis with the fast-start swimming speed as a response. The fast-start swimming speed was calculated as a distance that each tadpole swam after waving its tail three times and the time each tadpole took to wave its tail three times after the C-start. The possible explanatory variables were the time required for a tadpole to bend its body to swim away (C-start), the angle (in degrees) at which a tadpole bent its body for a C-start, body length, body width, tail length and tail width. Statistical analysis was performed using Minitab17 (Minitab Inc., State College, PA, USA).

Data on body weight, body length, and the ratio of tail length to body length in tadpoles are available in Table S3.

## Supplementary Material

Supplementary information
