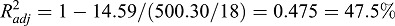# Correction: The constant threat from a non-native predator increases tail muscle and fast-start swimming performance in *Xenopus* tadpoles

**DOI:** 10.1242/bio.031443

**Published:** 2018-01-15

**Authors:** Tsukasa Mori, Yukio Yanagisawa, Yoichiro Kitani, Goshi Yamamoto, Naoko Goto-Inoue, Tadashi Kimura, Keiko Kashiwagi, Akihiko Kashiwagi

There was an error published in *Biology Open* (2017). **6**, 1726-1733 (doi: 10.1242/bio.029926).

An equation was incorrectly provided in the Table 2 legend. The corrected equation is below and the original article has been modified accordingly.