# Supplementary material for: The constant threat from a non-native predator increases tail muscle and fast-start swimming performance in Xenopus tadpoles
Source: Biol Open. 2017 Nov 15;6(11):1726–33. doi: 10.1242/bio.029926 (PMC5703619; doi:10.1242/bio.029926)
Supplement: Supplementary information [file biolopen-6-029926-s1.pdf]

Supplementary Information

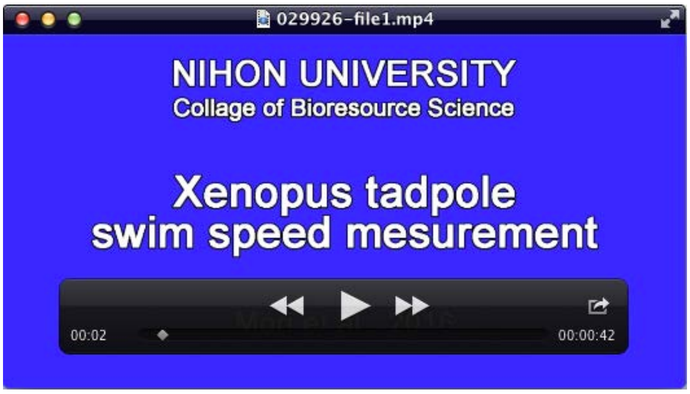

Movie 1. Swimming behavior of a tadpole in the electric chamber after delivery of electric shock filmed using a high-speed camera.

Table S1. Data on tail and muscle height from 50 tadpoles in each Exp 10 and Cont 10.

Click here to Download Table S1

Table S2. Data on swimming speed of tadpoles.

Click here to Download Table S2

Table S3. Data on body weight, body length, and the ratio of tail length to body length in tadpoles from each Exp 8 and Cont 8.

Click here to Download Table S3
